# Pd-Functionalized SnO_2_ Nanofibers Prepared by Shaddock Peels as Bio-Templates for High Gas Sensing Performance toward Butane

**DOI:** 10.3390/nano9010013

**Published:** 2018-12-23

**Authors:** Rongjun Zhao, Zhezhe Wang, Yue Yang, Xinxin Xing, Tong Zou, Zidong Wang, Ping Hong, Sijia Peng, Yude Wang

**Affiliations:** 1 School of Materials Science and Engineering, Yunnan University, Kunming 650091, China; Rjzhao0504@163.com (R.Z.); 13085343399@163.com (Z.W.); zoutong626@163.com (T.Z.); WangZiDongK@163.com (Z.W.); 18996659529@163.com (P.H.); sjpeng18@163.com (S.P.); 2 Department of Physics, Yunnan University, Kunming 650091, China; Yangyue_018@163.com (Y.Y.); xingxinxin@126.com (X.X.); 3 Key Lab of Quantum Information of Yunnan Province, Yunnan University, Kunming 650091, China

**Keywords:** bio-template, Pd-functionalized SnO_2_ nanofibers, gas sensor, butane

## Abstract

Pd-functionalized one-dimensional (1D) SnO_2_ nanostructures were synthesized via a facile hydrothermal method and shaddock peels were used as bio-templates to induce a 1D-fiber-like morphology into the gas sensing materials. The gas-sensing performances of sensors based on different ratios of Pd-functionalized SnO_2_ composites were measured. All results indicate that the sensor based on 5 mol % Pd-functionalized SnO_2_ composites exhibited significantly enhanced gas-sensing performances toward butane. With regard to pure SnO_2_, enhanced levels of gas response and selectivity were observed. With 5 mol % Pd-functionalized SnO_2_ composites, detection limits as low as 10 ppm with responses of 1.38 ± 0.26 were attained. Additionally, the sensor exhibited rapid response/recovery times (3.20/6.28 s) at 3000 ppm butane, good repeatability and long-term stability, demonstrating their potential in practical applications. The excellent gas-sensing performances are attributed to the unique one-dimensional morphology and the large internal surface area of sensing materials afforded using bio-templates, which provide more active sites for the reaction between butane molecules and adsorbed oxygen ions. The catalysis and “spillover effect” of Pd nanoparticles also play an important role in the sensing of butane gas as further discussed in the paper.

## 1. Introduction

Butane is a common gas, accounting for 70–80% in the mixtures of liquefied petroleum gas (LPG) [1]. Due to its properties of flammability, lack of color, distinct odor, and easy liquefaction or gasification, butane is widely used as a convenient fuel in lighters, residential heating, and industry [2]. Storage and transportation of butane are very important issues, which should not be ignored. Once butane gas leakages happen, these could be harmful to environment or even threaten human’s health. For instance, explosions could happen if butane concentrations exceed 1.6–8.5% by volume in air. More seriously, when the concentration of butane exceeds 800 ppm in air, symptoms will occur in the human body, such as dizziness, syncope, nausea, etc. [3]. Thus, precise monitoring of butane at low concentrations is beneficial to prevent accidental events. In other words, it is necessary to develop gas sensors with good gas-sensing performances (such as high gas response, low detection limit, good selectivity, rapid response/recovery, low operating temperature and good stability) to monitor and detect butane gas at real-time. 

Up to now, different types of nanostructured materials have attracted attention as gas sensing materials, including SnO_2_ [4], ZnO [5], polypyrrole [6], TiO_2_ [2,7], SnO_2_/In_2_O_3_ nanocomposites [8], nanostructured conjugated polymers [9], SnO_2_/carbon nanofiber composites [10], nanostructured polyaniline/gold composites [11], etc. Among those, SnO_2_, a typical n-type wide band gap semiconductor (*E*_g_ = 3.6 eV at 300 K), is widely used in gas sensors due to its unique physical and chemical properties and good thermal stability. On the other hand, compared with other types of sensors, for example, mass spectrometry (MS), gas chromatography (GC) [12], etc., SnO_2_ based gas sensors received more and more attention in practical applications because of their facile preparation, low-cost, non-toxicity, excellent sensing performances and simple operation. The principle of semiconductor-based gas sensors is mainly based on changes in charge carrier concentration, leading to resistance changes when the gas sensor is exposed to air or test gases. The gas-sensing process mainly includes adsorption, electron transfer and desorption of test gas molecules on the surface of the sensing material [13]. Generally, the sensor properties are strongly affected by the structure, morphology, surface area, crystalline size and materials compositions. In the past few years, SnO_2_ nanomaterials with different structures have been widely studied, and used as sensor materials to detect various harmful and flammable gases. For example, Xi et al. prepared SnO_2_ nanoparticles via a homogeneous precipitation ethanol-thermal method and used these particles as a sensing material to detect ethanol [14]. SnO_2_ nanowires were synthesized by Wang et al. and were applied in gas sensors [15]. Wang’s group reported on SnO_2_ microspheres, prepared by a hydrothermal route, and used them as sensing material for formaldehyde detection. In addition, they also prepared raspberry-like SnO_2_ hollow nanostructures by a template-free hydrothermal method and used them to detect n-butanol gas [16,17]. Zhou et al. prepared 3D flower-like SnO_2_ hierarchical structures with enhanced ethanol gas-sensing performances [18]. However, similar to most metal oxides, pristine SnO_2_ based sensors suffer from some problems, such as low response, poor selectivity and long-term stability as well as high working temperatures, which usually hinder their use in practical applications. To ameliorate these problems, many researchers performed work devoted to improving gas-sensing performances by doping noble metals such as Au, Ag, Pd, and Pt [19,20,21,22], or forming compounds with other metal oxides [23] or novel synergistic structures with other elements [24].

More recently, biomaterials have been used as structural templates to prepare inorganic macro/nanomaterials with morphologies following these natural templates. Particularly, many novel structural SnO_2_ materials are prepared via the assistance of different bio-templates to form sensor materials. Zhang et al. prepared single porous SnO_2_ microtubes using Papiliomaacki bristles as templates, which enabled enhanced gas-sensing properties toward ethanol, formaldehyde and ammonia [25]. Zhu et al. used cottons as bio-templates to prepare SnO_2_ nanotubular materials, which showed good gas-sensing performances towards acetone [26]. Song et al. also prepared bioinspired hierarchical SnO_2_ scaffolds using ripe pollen grains as bio-templates [27]. In this way, novel structural materials could be prepared simply from different bio-templates by reproducing the structures of these natural biomaterials, which were also used as sensor materials, exhibiting excellent gas-sensing performances. It is worth mentioning that bio-templates play an important role in the design and preparation of new materials because they are low-cost, environmentally friendly and easy to prepare. In general, 1D nanostructural materials exhibit excellent gas-sensing properties due to large surface areas. Thus far, most 1D nanomaterials are prepared via chemical routes [15] or by electrospinning technology [28]. More recently, the grapefruit exocarp was used as a template to prepare SnO_2_ materials by Wang et al. [13]. After soaking the templates in SnCl_4_ solution and after calcination, they obtained SnO_2_ with a porous hierarchical morphology, which followed the morphology of the natural bio-templates. However, 1D structural SnO_2_ fibers were synthesized using shaddock peels as bio-templates and functionalized by Pd via a facile hydrothermal route for the first time in this work.

In this work, different (the molar ratio of Pd to Sn: 0, 1, 3, 5, 7 mol %) Pd-functionalized SnO_2_ nanofibers were prepared by using shaddock peels as bio-templates and urea as additive via a facile hydrothermal route and by annealing in air. The as-prepared Pd-functionalized SnO_2_ nanofibers were used as sensor materials to detect different flammable gases, including butane, methane, carbon monoxide and hydrogen. The 5 mol % Pd-functionalized SnO_2_ nanofiber-based sensors exhibited distinctly enhanced butane gas-sensing performances compared to other as-prepared Pd-SnO_2_ composites. The improved gas-sensing performances, such as high response, low operating temperature, fast response/recovery and good stability, may be mainly attributed to the introduction of Pd and increased surface areas. Functionalization with noble metals can promote the reaction between butane molecules and adsorbed oxygen ions on the surface of SnO_2_. Results also demonstrate that Pd-functionalized SnO_2_ nanofibers prepared using shaddock peels as bio-templates may be a promising sensing material in butane detection.

## 2. Materials and Methods

### 2.1. Synthesis of Pd-Functionalized SnO_2_ Nanofibers

All chemical reagents in the experiment were analytic grade and obtained from commercial sources without any further purification, including tin chloride pentahydrate (SnCl_4_·5H_2_O), urea (CO(NH_2_)_2_), palladium chloride (PdCl_2_) and absolute ethanol. Shaddock peels were obtained from a local fruit store.

First, the white parts were separated from the shaddock peels and divided into many small bulks. Then, they were washed in distilled water and absolute ethanol several times. In addition, the shaddock peels were ultrasonically treated for 1 h and dried at 60 °C. Pd-functionalized SnO_2_ composites were prepared via a facile hydrothermal route by using shaddock peels as bio-templates. Typically, 10 mmol SnCl_4_·5H_2_O were dissolved in a mixture of 40 mL distilled water and absolute ethanol (volume ratio of distilled water and ethanol is 1:1) under magnetic stirring. Subsequently, appropriate amounts of PdCl_2_ (molar ratios of Pd to Sn: 1, 3, 5 and 7 mol %) was added into the mixture under stirring until dissolved completely. Then, 1 g dried shaddock peels and 0.2404 g urea were added into the mixture under continuous stirring for another 4 h. Thereafter, the mixture was ultrasonically treated for 30 min and then transferred into a Teflon-lined stainless-steel autoclave and treated at 150 °C for 12 h in a lab oven. After the shaddock peels had been carbonized under hydrothermal treatment, hydroxyl (–OH) groups are expected to be on the surface which combine with Sn^4+^. After the autoclave cooled down to room temperature naturally, the resultant precipitates were centrifuged, washed in distilled water and absolute ethanol several times, respectively, and dried at 60 °C in air. Finally, the precipitates were annealed in a furnace at a heating rate of 2 °C/min up to a temperature of 550 °C, and kept constant there for 2 h in air to remove the bio-templates. After the furnace was allowed to cool down to room temperature, Pd-functionalized SnO_2_ powders were obtained. In the case of pure SnO_2_, we followed the same prepared procedure except for adding PdCl_2_.

### 2.2. Characterization of As-Prepared Products

The phase identification and structures of the as-prepared Pd-functionalized SnO_2_ samples were investigated by powder X-ray diffraction (XRD, TRIII, Rigaku Corporation, Tokyo, Japan) using Cu Kα radiation (λ = 1.54056 Å). All measurements were carried out in the 2θ range from 20° to 80° in steps of 0.02°. The morphology and microstructure of the as-prepared samples were observed by field-emission scanning electron microscopy (FESEM) (FEI NOVA NANOSEM 450, FEI Company, Hillsboro, OR, USA). In addition, transmission electron microscopy (TEM) and high-resolution transmission electron microscopy (HRTEM) were used to investigate into more detailed the microstructural features of Pd-functionalized SnO_2_, which were performed on a JEM-2100 (JEOL, Tokyo, Japan) at operating acceleration voltages of 200 kV. The surface chemistries and chemical compositions were analyzed by X-ray photoelectron spectroscopy (XPS) (Thermo Fisher Scientific Company, Waltham, MA, USA) with Al Kα X-ray radiation at 12 kV. The specific surface areas of as-prepared samples were obtained from Brunauer–Emmett–Teller (BET) nitrogen adsorption isotherm measured at 77.3 K with a Micrometrictics ASAP 2010 automated sorption analyzer (Norcross, GA, USA). At the same time, the distributions of pore size were obtained using the Barrett–Joyner–Halenda (BJH) method from the desorption isothermal.

### 2.3. Fabrication and Measurement of Gas Sensors

Gas sensors were fabricated using different (0, 1, 3, 5, and 7 mol %) Pd-functionalized SnO_2_ samples as sensing materials by employing the indirect heating method [29]. Typically, a certain amount of as-prepared powders was mixed with some drops of distilled water to form a homogeneous paste. Subsequently, the paste was coated onto the outside surface of an alumina ceramic tube (4 mm in length, 1.2 mm in external diameter and 0.8 mm in internal diameter, respectively) with a pair of Au electrodes and four platinum wires. The thickness of sensing material on the surface of alumina tube amounted to about 0.6 mm. Then, the coated sensors were dried at 120 °C for 1 h and calcined at 400 °C for 1 h to guarantee a good contact between Au electrodes and sensing materials. A Ni-Cr alloy coil (~28 Ω) was inserted in the alumina tube to control the operating temperature via adjusting the heating voltage. In addition, to improve the stability of the sensor, all sensors were aged at the operating temperature of 350 °C for 150 h in dry air. 

The gas-sensing performances of sensors were measured by a WS-30A (Weisheng Instrument Co., Zhengzhou, China) commercial gas-sensing measurement system [30]. The measurements were carried out using a static process. During the testing process, the desired amount of target gas was injected into the test chamber with a volume of 18 L. Then, target gas mixed with fresh air immediately by two fans installed in the test chamber. After the sensor resistance had stabilized in the test gas atmosphere, the upper cover of the chamber was removed and the sensor started to recover in fresh air. It is well known that, as a typical n-type semiconductor based gas sensor, the response (β) can be defined as the ratio of *R*_a_ to *R*_g_, where *R*_a_ and *R*_g_ are the resistances of the sensor in air and in test gas, respectively. The response and recovery times are defined as the time required to achieve 90% of the initial equilibrium values.

## 3. Results and Discussion

### 3.1. Structural and Morphological Characteristics

**Figure 1:**
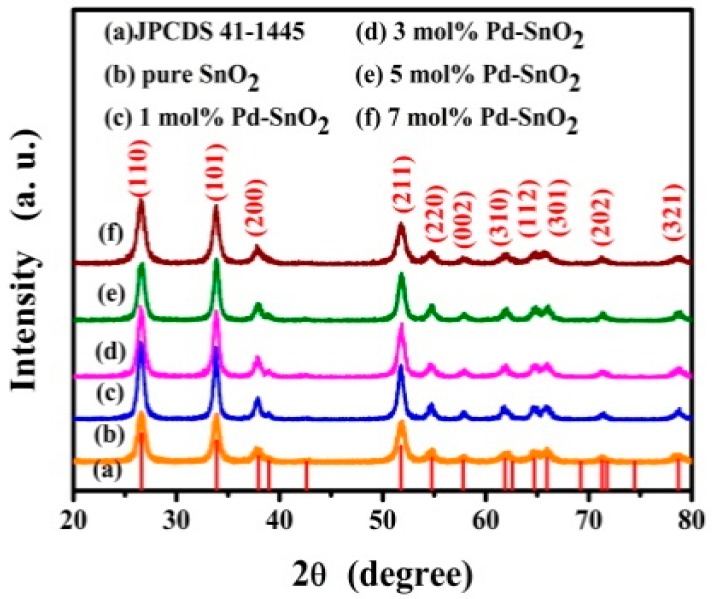
XRD patterns of as-prepared samples with different Pd contents.

XRD was carried out to identify the crystal structure and phase compositions of the as-prepared different Pd-functionalized SnO_2_ samples (0, 1, 3, 5 and 7 mol %). In Figure 1, one can see that all diffraction peaks can be clearly assigned to the tetragonal rutile crystal phase of SnO_2_ (JCPDS: 41-1445), space group P4_2_/mnm (136) for all samples. No diffraction peaks arising from impurities can be observed. In the differently Pd-functionalized SnO_2_ samples (0, 1, 3, 5 and 7 mol %), no characteristic peaks arising from Pd or PdO were observed, as these concentrations are probably too low to be detectable by XRD. The crystallite sizes (D) of different samples were estimated by the well-known Scherer equation: D = Kλ/Bcosθ, where K is a constant taken as 0.89, λ is the X-ray wavelength, θ is the diffraction angle and B the full width at half maximum [31]. The corresponding results are listed in Table S1 (see Supplementary Materials).

**Figure 2:**
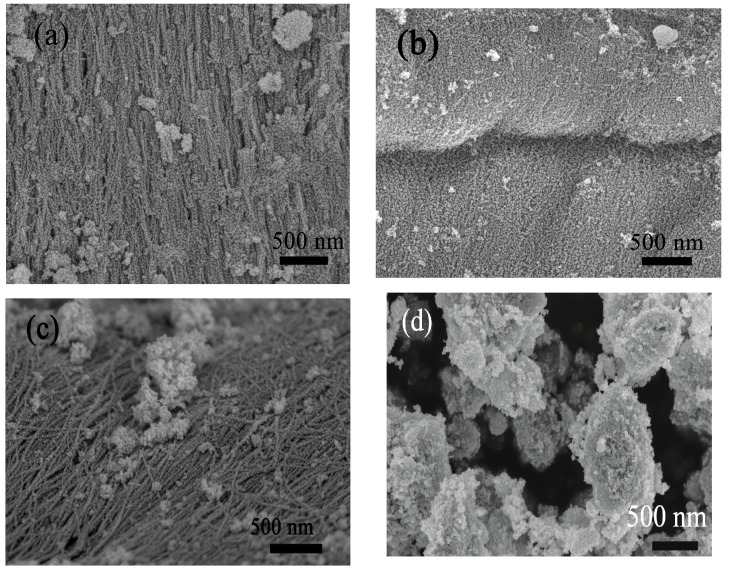
Field-emission scanning electron microscopy (FESEM) images of as-prepared 5 mol% Pd-functionalized SnO_2_ nanofibers containing shaddock peels before (**a**) and after calcination (**b**), respectively; (**c**) FESEM image of 3 mol % Pd-functionalized SnO_2_ nanofibers after calcination; and (**d**) FESEM image of a control sample prepared without adding shaddock peels after calcination.

Figure 2a,b shows the morphologies of a SnO_2_ sample functionalized with 5 mol % Pd as revealed by FESEM. Figure 2a shows the FESEM image of 5 mol % Pd-functionalized SnO_2_ sample before annealing. This as-prepared sample features a 1D fiber-like structure composed of plenty of nanoparticles. Particularly, the SnO_2_ particles are arranged in an orderly manner. This first picture shows that the hydrothermal process preserves the fiber morphology of the original bio-templates. Figure 2b shows the state after annealing at 550 °C. One can see that the calcined sample retains the 1D fiber-like structure composed of many small nanoparticles. The morphologies of other samples with different Pd contents (0, 1, 3 and 7 mol %) are similar. This fact is demonstrated by Figure 2c, which shows the morphology of 3 mol % Pd-functionalized SnO_2_ sample after annealing. Figure 2d shows the morphology of a 5 mol % Pd-functionalized SnO_2_ sample that had been prepared without adding shaddock peels. It displays an irregular array of particles with different sizes, and no 1D fiber-like structure. Altogether the results demonstrate that 1D Pd-functionalized SnO_2_ nanofibers were successfully synthesized by a facile hydrothermal route using shaddock peels as a scaffolding agent to transfer the 1D nanofiber morphology into the inorganic metal oxide domain.

**Figure 3:**
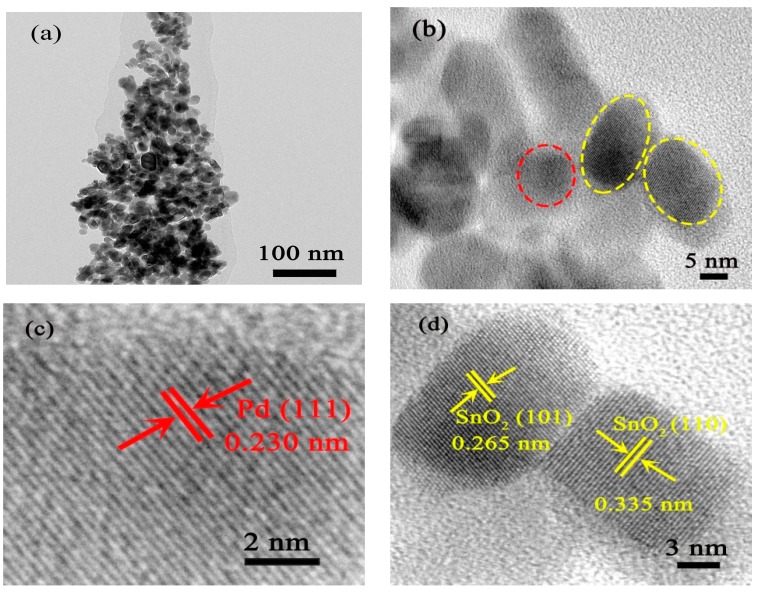
Transmission electron microscopy (TEM) (**a**) and high-resolution transmission electron microscopy (HRTEM) (**b–d**) images of as-prepared 5 mol% Pd-functionalized SnO_2_ nanofibers after annealing.

More detailed microstructural information about all samples was studied by TEM and HRTEM. Figure 3 displays TEM images of as-prepared 5 mol % Pd-functionalized SnO_2_ nanofibers. In Figure 3a, it can be observed that 1D fiber-like 5 mol % Pd-functionalized SnO_2_ consists of numerous nanoparticles with irregular shapes, aggregated along a single dimension, which further supports our FESEM results. The corresponding HRTEM images of 5 mol% Pd-functionalized SnO_2_ are displayed in Figure 3b–d. Figure 3b confirms that these nanoparticles possess irregular shapes and different grain sizes. Obviously, Pd nanoparticles can be found, embedded within SnO_2_ nanoparticles. These special locations are marked with red and yellow dashed lines, which correspond to Pd and SnO_2_ nanoparticles, respectively. In addition, the clear lattice fringes demonstrate that a good crystallization of the as-prepared samples has occurred during calcination. As shown in Figure 3c, the inter-planar distance of 0.230 nm matches well with the (111) plane of the Pd phase [8]. On the other hand, the lattice fringes at *d* = 0.265 nm and 0.335 nm correspond to the (101) and (110) planes of the tetragonal rutile structural of SnO_2_, respectively (Figure 3d). Additionally, the sizes of Pd particles for all samples were estimated from TEM analysis and are listed in Table S1. All results indicate that the Pd-functionalized SnO_2_ nanofibers were successfully prepared using shaddock peels as bio-templates and are composed of many small nanoparticles.

To obtain the specific surface areas and the corresponding pore size distributions of the as-prepared samples with different Pd contents, N_2_ adsorption–desorption measurements were performed, as shown in Figure S1. In Figure S1a–e, distinct hysteresis loops can be observed at high relative pressures in the different samples, which can be ascribed to a type IV isotherms with type H_3_ hysteresis loops and demonstrate the existence of pores. Besides, the pore size distributions were obtained using the BJH method from the desorption branch of the isotherm, which showed microporous structure with different pore sizes (see the inset of Figure S1a–e). In addition, the specific surface areas of the differently Pd functionalized SnO_2_ samples were calculated using the BET method [32]. The values are shown in Table S1. In Table S1, it is evident that the specific surface areas of Pd-functionalized SnO_2_ nanofibers are significantly increased with regard to pure SnO_2_, which is likely an essential factor for enhanced gas-sensing performance. Generally, large internal surface areas and a high level of porosity are important assets of gas sensor materials, as these can provide more active sites on the surfaces for gas adsorption, chemical reactions, gas desorption, gas molecules diffusion and electrons transfer.

XPS was carried out to further investigate the compositions and chemical states of all samples. The results demonstrate that all samples only contain O-, Sn-, and Pd-related peaks, which reveals the high purity of the as-prepared samples. This fact is illustrated in Figure S2, which shows the spectra of 5 mol % Pd-functionalized SnO_2_ nanofibers. Figure S2a shows the survey spectrum of 5 mol % Pd-functionalized SnO_2_ nanofibers. In Figure S2b, one can find that the XPS spectrum of Sn 3d is divided into two symmetrical peaks of Sn 3d_3/2_ and Sn 3d_5/2_ at binding energies of 496.68 and 486.23 eV, respectively. In addition, the peak separation of 8.43 eV between both peaks is in agreement with the energy reported for SnO_2_, which correspond to the Sn 3d binding energy of Sn^4+^ [29]. The O 1s spectrum is shown in Figure S2c. Obviously, two distinct peaks with binding energies of 530.18 eV and 531.59 eV correspond to the lattice oxygen (O_lat_) and adsorbed oxygen O*_x_*^−^ (O_2ads_^−^, O_ads_^−^ or O_ad__s_^2−^), respectively [33]. O_lat_ are attributed to oxygen atoms inside the crystal lattice of SnO_2_. These are thought to be very stable and therefore cannot react with target gas molecules, thus O_lat_ cannot impact the gas-sensing performance. The O*_x_*^−^ (O_2ads_^−^, O_ads_^−^ or O_ad__s_^2−^) species, on the other hand, are attributed to chemically adsorbed oxygen ions on the surface, which can react with target gases and therefore play an important role in the gas sensing process [8]. The concentration ratios of adsorbed oxygen and lattice oxygen for different samples were analyzed, as shown in Table S2. The ratio of adsorbed oxygen increases with increasing Pd content, which is likely due to the enhanced internal surface area in Pd-functionalized samples. The increased amount of adsorbed oxygen on the sensing surface layer is helpful to improve the gas-sensing properties. Moreover, the high-resolution XPS spectra of Pd 3d are shown in Figure S2d; the state of Pd 3d consists of spin-orbitals of Pd 3d_3/2_ and Pd 3d_5/2_ at binding energies of 341.72 and 336.42 eV, respectively. The difference of binding energies between Pd 3d_3/2_ and Pd 3d_5/2_ is 5.30 eV, which is good agreement with reported values, indicating the presence of Pd nanoparticles in the 5 mol % Pd-functionalized SnO_2_ nanofibers [34].

### 3.2. Gas-Sensing Properties of Pd-Functionalized SnO_2_

To further investigate the application potential of the prepared materials, sensors based on Pd-functionalized SnO_2_ nanofibers with different Pd contents (0, 1, 3, 5 and 7 mol %) were fabricated. Gas-sensing performances were measured by a commercial gas-sensor test system. Furthermore, all measurements were conducted three times and data are reported as averages ± standard deviation in this work. 

**Figure 4:**
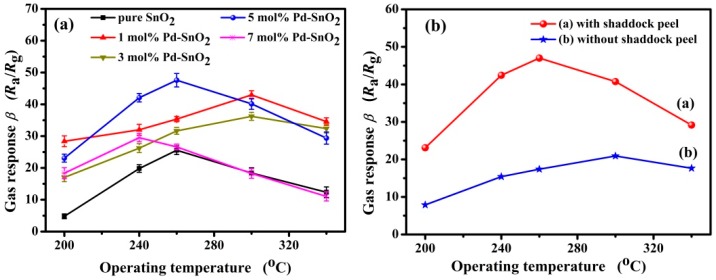
(**a**) Gas response as a function of temperature toward 3000 ppm butane of sensors prepared from SnO_2_, functionalized with different amounts of Pd; and (**b**) temperature dependence of gas response of two sensors prepared from SnO_2_ functionalized with 5% Pd, one formed with (red) and the other without (blue) addition of shaddock peels.

In general, the gas responses of the sensors are strongly affected by the operating temperature, as it affects the adsorption and desorption of gases on the surface of the sensing materials. The responses of gas sensors based on different contents of Pd-functionalized SnO_2_ nanofibers were measured toward 3000 ppm butane gas in the operating temperature range extending from 200 to 340 °C. In Figure 4a, it is obvious that the responses of all sensors are highly dependent on the operating temperature. All curves exhibit a similar tendency, with the response first increasing with increasing operating temperature, going through a maximum at an optimum operating temperature, and dropping off at even higher temperatures. In Figure 4a, one can see that the introduction of noble metal Pd not only affects the working temperature of sensors, but it also improves the gas response toward butane as compared to pristine SnO_2_. Among all sensors investigated, the one based on 5 mol % Pd-functionalized SnO_2_ nanofibers showed the highest gas response of 47.58 ± 2.11 to 3000 ppm butane at an operating temperature of 260 °C. The responses of sensors based on pristine SnO_2_, 1, 3 and 7 mol % Pd-functionalized SnO_2_ nanofibers are 25.57 ± 1.36, 35.36 ± 0.85, 31.64 ± 1.07 and 26.60 ± 0.87, respectively, as measured at the same operating temperature. The enhanced response may be attributed to the increased surface areas in Pd-functionalized materials and the catalytic effect of Pd, which accelerates the reaction between adsorbed oxygen and butane molecules. That these favorable properties are indeed due to the scaffolding afforded by the introduction of shaddock peels is illustrated in Figure 4b. In this figure, two gas sensors are compared, both formed from SnO_2_ material functionalized with 5 mol % Pd. Whereas the first sensor was prepared in the usual way with additions of shaddock peels, the other was prepared from the same kind of SnO_2_ material but without additions of shaddock peels. Figure 4b clearly shows that the sensor prepared with shaddock peels exhibits a significantly higher butane response at lower operating temperature when compared to the reference sensor prepared without shaddock peels.

**Figure 5:**
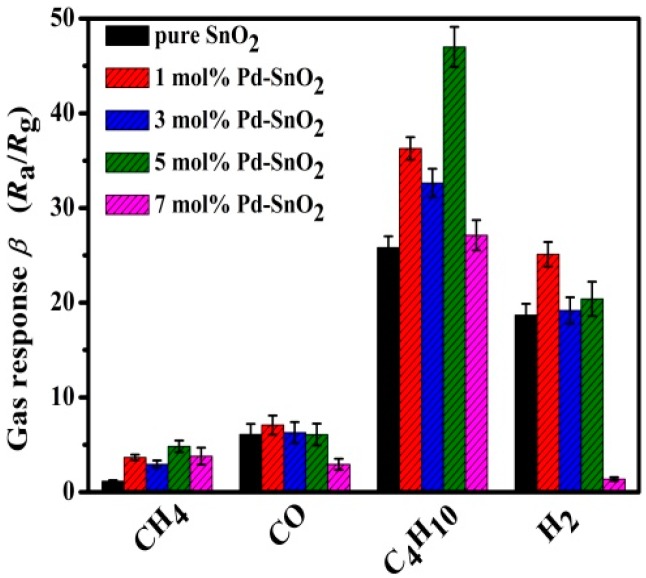
Gas responses of sensors based on Pd-functionalized SnO_2_ nanofibers with different Pd contents to various flammable gases, all applied at a concentration of 3000 ppm and as measured at an operating temperature of 260 °C.

Selectivity is another important criterion to evaluate the performance of gas sensors in practical applications. Therefore, gas responses toward 3000 ppm of various flammable gases (including butane (C_4_H_10_), methane (CH_4_), hydrogen (H_2_) and carbon monoxide (CO)) were measured at the optimal operating temperature of 260 °C. Figure 5 shows that the responses of sensors based on Pd-functionalized SnO_2_ nanofibers are higher than those of sensor based on pristine SnO_2_ under same test conditions. In particularly, the 5 mol% Pd-functionalized SnO_2_ nanofiber-based sensor showed the highest response of 47.58 ± 2.11 to 3000 ppm butane at 260 °C, while the responses to methane, hydrogen and carbon monoxide are about 4.87 ± 0.62, 20.40 ± 1.81 and 6.08 ± 1.14, respectively. This enhanced response can be attributed to the catalytic activity of Pd and the larger internal surface areas of the Pd-functionalized SnO_2._ The species-dependent response to different gases is likely due to different activation energies (E_a_) for catalytic combustion and to the influence of diffusion-reaction phenomena in these highly porous materials [35,36]. In the case of reducing gases, it is well known that the relative strength of C–H bonds can significantly affect the response and selectivity of gas sensors. If hydrocarbon molecules contain more carbon atoms, they are expected to dissociate more easily. As the C–H bond dissociation energy for butane (425 KJ/mol) is lower than that of methane (436 KJ/mol) [37], methane is more stable and thus more difficult to dissociate via reactions with adsorbed oxygen. As oxidation reactions with H_2_ or CO are more difficult to conduct under same test conditions, butane can more quickly react with adsorbed oxygen and diffuse into sensing layer. Thus, the sensor exhibits a higher response to butane and a certain degree of selectivity to this gas. Therefore, the 5 mol% Pd-functionalized SnO_2_ nanofiber-based sensor can distinguish butane gas, particularly when such sensors are used in arrays with sensors that exhibit dissimilar cross sensitivity profiles obviously among different flammable gases. This point is further illustrated by the data in Table 1, which lists the butane sensing performances of sensors based on various metal oxides. Obviously, the sensor based on 5 mol% Pd-functionalized SnO_2_ nanofibers shows better gas-sensing performances toward butane than the other metal oxide gas sensors. Although the responses of sensors based on Pt-AZO nanosheets (56.52) [5] and 7 mol% Pd-SnO_2_/In_2_O_3_ (71.28) [8] are higher than those reported for the 5 mol% Pd-functionalized SnO_2_ nanofiber-based sensor (47.58 ± 2.11) with concentration of 3000 ppm, the latter exhibits faster response/recovery times (3.20 s/6.28 s) at lower sensor operating temperatures (260 °C). Hence, all results demonstrate that the sensor based on 5 mol% Pd-functionalized SnO_2_ nanofibers has an encouraging butane sensing performance.

**Figure 6:**
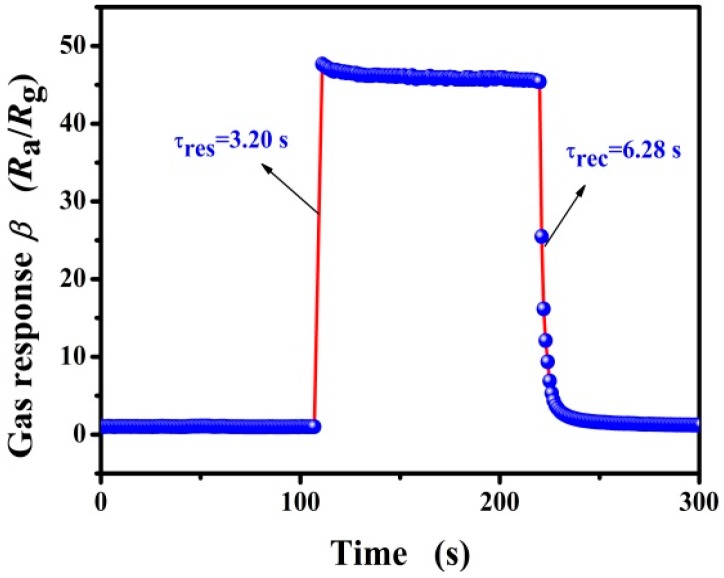
The typical response and recovery times of the sensor based on 5 mol % Pd-functionalized SnO_2_ nanofibers at the optimal operating temperature of 260 °C for 3000 ppm butane.

Response and recovery times are important sensor performance parameters. These times are defined as times taken by the response values to achieve 90% of their final equilibrium values [41]. The response and recovery characteristics of the sensor based on 5 mol % Pd-functionalized SnO_2_ nanofibers were measured at 260 °C and under exposure of 3000 ppm of butane (Figure 6). The response and recovery times are about 3.20 s and 6.28 s, respectively. Obviously, the sensor exhibits rapid response and recovery times when exposed to butane gas and fresh air. In addition, the remarkably shorter response and recovery times may be attributed to the catalytic activity of the Pd nanoparticles, which are likely to accelerate the reaction between adsorbed oxygen ions and target gas molecules on the internals surfaces of the sensing layer [19,42].

Reproducibility is another important criterion [43]. To evaluate the reliability of the developed gas sensors, the evolution of the gas responses during six cycles was tested toward 3000 ppm butane at 260 °C. Figure S3 displays the reproducibility of a sensor based on 5 mol% Pd-functionalized SnO_2_ nanofibers. Obviously, the response increases up to a high value after butane is injected and maintains it as long as the sensor is exposed to butane. Thereafter, the sensor response quickly recovers to baseline when the sensor is exposed to fresh air again. It is obvious that the sensor maintains the initial response without any clear indication of change after six cycles of testing at an average response value of about 46.84 ± 0.46. This result indicates that the sensor based on 5 mol % Pd-functionalized SnO_2_ nanofibers shows a good reproducibility under real-time monitoring conditions of butane gas. 

**Figure 7:**
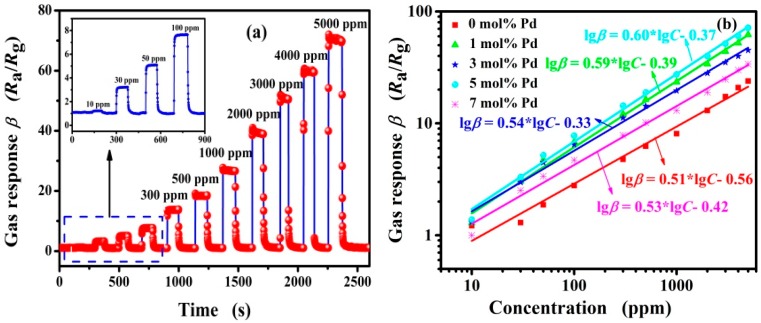
(**a**) The dynamic response/recovery curves of the sensor based on 5 mol % Pd-functionalized SnO_2_ nanofibers to different butane concentrations from 10 to 5000 ppm at 260 °C; and (**b**) the variation of sensors based on (0, 1, 3, 5 and 7 mol %) Pd-functionalized SnO_2_ nanofibers to butane with concentrations range from 10 to 5000 ppm at 260 °C.

To further investigate the potential application of 5 mol % Pd-functionalized SnO_2_ nanofibers as a reliable gas-sensing material, the typical dynamic response/recovery characteristics were studied toward butane in the concentration range from 10 to 5000 ppm at 260 °C. In Figure 7a, it can be seen that the response increases with increasing butane concentration from 10 to 5000 ppm at 260 °C. The result demonstrates that the sensor based on 5 mol% Pd-functionalized SnO_2_ nanofibers can detect butane over a wide concentration range with a detection limit as low as 10 ppm, at which it shows a response of 1.38 ± 0.26. Therefore, 5 mol % Pd-functionalized SnO_2_ nanofibers appear to be a promising sensing material with a high and reliable response and a low detection limit to butane. Figure 7b depicts the relationship curves between the response and butane concentration of different sensors at 260 °C. Obviously, the Pd content has a large influence on the response of sensors to detect butane. The curves between the responses and concentrations exhibit a good linear trend to different sensors and the relationship can be described as lgβ = k × lg*C* + B, where β is the gas response of sensor, *C* is the concentration of butane, and k and B are different constants, respectively. This good linear relationship indicates that the sensor has potential application for butane detection in a wide concentration range and in real-time. 

In practical applications, stability is another vital parameter of the gas-sensing performance of a sensor. Thus, the 5 mol % Pd-functionalized SnO_2_ nanofiber-based sensor was exposed to 3000 ppm butane at 260 °C for 30 days to test the stability of the sensor and the results are shown in Figure S4. It can be noted that the response values exhibit a small variation during the test period, and the average response value is about 47.25 ± 1.93. This illustrates that the sensor based on 5 mol% Pd-functionalized SnO_2_ nanofibers shows a good long-term stability for butane detection.

### 3.3. Gas-Sensing Mechanism

**Figure 8:**
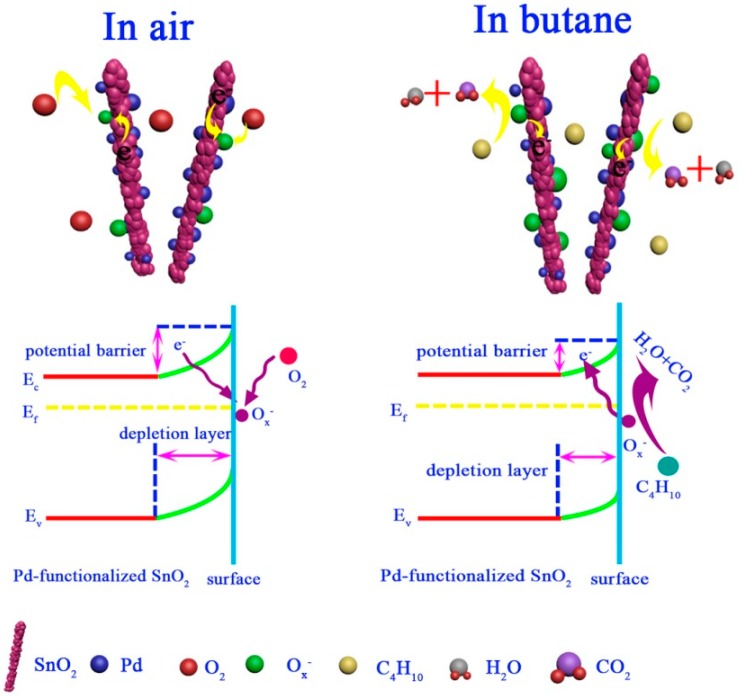
Schematic illustration of the gas-sensing mechanism toward butane and the energy band structure of the sensor based on 5 mol % Pd-functionalized SnO_2_ nanofibers in air and butane.

According to the above results, the sensing mechanism of metal oxide semiconductor-based gas sensors can be interpreted in terms of the depletion layer model [44,45]. The illustration of the gas-sensing mechanism and the changes in the band energy structure for 5 mol % Pd-functionalized SnO_2_ nanofibers in air or butane are shown in Figure 8. Gas adsorption, electron transfer and desorption are regarded as key processes during gas detection [13], which results in a change in carrier concentration and a sensor resistance increase or decrease. In more detail, when the sensor is exposed to air, oxygen molecules are adsorbed onto the surface of the Pd-functionalized SnO_2_ nanofibers, thereby capturing electrons from the conduction band of materials and forming oxygen ion species O*_x_*^−^ (O_2ads_^−^, O_ads_^−^ or O_ads_^2−^). In this way, a thick electron depletion is formed beneath the surface of the sensing layer. Therefore, the sensors based on Pd-functionalized SnO_2_ nanofibers show a higher resistance due to the lowered charge carrier concentration in the conduction band [46,47]. At the same time, the potential barrier also increased as the following reactions happen [48]:

O_2(gas)_ → O_2ads_ (1)

O_2ads_ + e^−^ → O_2ads_^−^ (2)

O_2ads_^−^ + e^−^ → 2O_ads_^−^ (3)

O_ads_^−^ + e^−^ → O_ads_^2−^ (4)

Once the 5 mol % Pd-functionalized SnO_2_ nanofiber-based sensor is exposed to a reducing gas, such as butane, butane molecules will react with the co-adsorbed oxygen ions on the surfaces of the metal oxide to form CO_2_ and H_2_O, which causes the trapped electrons to be released back to the conduction band of the semiconductor [48]. The increased carrier concentration in the conduction band, in turn, causes a drop in the sensor resistance. At the same time, the thickness of the depletion layers is decreased and the heights of potential barriers are reduced. This process can be described by the following reactions [2]:

C_4_H_10(gas)_ → C_4_H_10(ads)_ (5)

C_4_H_10(ads)_ + 13O_ads_^−^ → 4CO_2_ + 5H_2_O +13e^−^ (6)

C_4_H_10(ads)_ + 13O_ads_^2−^ → 4CO_2_ + 5H_2_O +26e^−^ (7)

According to the abovementioned results and analysis, the enhanced butane gas-sensing performances of sensor based on 5 mol % Pd-functionalized SnO_2_ nanofibers can be attributed to the following facts: Firstly, the 5 mol % Pd-functionalized SnO_2_ sample prepared by using shaddock peel as bio-template shows a unique morphology with 1D nanofibers composed of numerous small nanoparticles. This special nano-morphology leads to a large specific surface area (44 m^2^/g), which provides more active sites for the reaction between butane molecules and adsorbed oxygen ions. The as-prepared 1D structure also shows porosity, which was confirmed by N_2_ adsorption–desorption measurements (Figure S1d). The existence of such a nanoporous structure appears to be beneficial to the diffusion of butane gas molecules through the interior of the gas sensing layers, thus leading to good gas-sensing performance. Moreover, the existence of adsorbed oxygen also plays an important role in gas sensor, as measured by XPS (Figure S2), which reacted with butane gas molecules and resulted in the response of sensor according to the change of resistance in air or butane, respectively.

Appropriate noble metals, such as Pd, Pt, Ag, Au, etc., are widely used in the sensor technology to enhance gas-sensing performances [42,49,50]. In this work, the much better butane sensing performance for 5 mol % Pd-functionalized SnO_2_ nanofiber-based sensor can be attributed to the introduction of Pd, which may due to the chemical sensitization and electronic sensitization of noble metal catalyst [34,51]. After Pd nanoparticles functionalized, the oxygen species can be adsorbed on the surface of Pd-SnO_2_ composites more easily because of the “spillover effect” [52]. Besides, the Pd nanoparticles act as a catalyst during the detection, as it can accelerate the dissociation of oxygen molecules and promote the reaction rate between adsorbed oxygen ions and butane on the surface of Pd-SnO_2_ composite with regard to pure SnO_2_ [42]. In addition, the results also reveal that excess functionalization by Pd could lead to a reduction in gas response, which may be due to a reduction in the size of effective active areas of inside the sensing materials [53]. Therefore, all results demonstrate that a higher specific surface area and the appropriate Pd functionalization highly affect the gas-sensing performances toward butane.

## 4. Conclusions

Different (0, 1, 3, 5, 7 mol %) Pd-functionalized SnO_2_ nanofibers were prepared via a facile hydrothermal method, using shaddock peels as bio-templates. After infiltration of the bio-templates with inorganic chemicals, an annealing process in air was carried out, in which the infiltrated chemicals reacted and sintered into an inorganic body of metal oxide material, while the scaffolding bio-templates were converted into CO_2_ and H_2_O. In this way, the 1D-fiber morphology of the bio-templates was successfully transferred into the inorganic domain. The structure, morphology, chemical compositions and specific surface area of these sintered porous materials were investigated by different characterization methods. The results show that the so-formed metal oxide materials are composed of many small nanoparticles, which were sintered into 1D fiber-like structures. Additionally, the gas-sensing performances of sensors based on different Pd-functionalized SnO_2_ were measured using a gas static test system. Among all sensors investigated, the 5 mol% Pd-functionalized SnO_2_ sensor showed the best gas-sensing performance towards butane. The gas-sensing performance at the optimal operating temperature of 260 °C featured high response, moderate selectivity, good repeatability, long-term stability, rapid response/recovery and a wide detection range. Detection limits as low as 10 ppm butane with the response of 1.38 ± 0.26 were observed. The significant enhancement of the gas-response to butane is not only attributed to the unique structure with 1D fiber-like and large specific surface area (44 m^2^/g), but also importantly due to the functionalization by means of Pd nanoparticles. Furthermore, the results indicate that the appropriate Pd-functionalized SnO_2_ possesses excellent gas-sensing characters, thus is a potential sensor material for butane detection in practical applications.

## Figures and Tables

**Table 1:** Comparison of gas-sensing performances of sensors based on various nanostructural materials toward butane.

Materials	*C* (ppm)	OT (°C)	τ_res_/τ_rec_ (s)	β	Ref.
W-doped TiO_2_ nanoparticle	3000	420	2./12	17.80	[2]
Hollow ZnSnO_3_	500	380	0.3/0.65	5.79	[3]
Pt-AZO nanosheets	3000	200	31/28	56.52	[5]
7.5 mol% Pd-TiO_2_	3000	340	13/8	33.93	[7]
SnO_2_/In_2_O_3_	3000	320	3.40/5.82	29.27	[8]
7 at% Pd-SnO_2_/In_2_O_3_	3000	320	3.51/7.86	71.28	[8]
SnO_2_ quantum dots	100	400	3/8	5.10	[38]
γ-Fe_2_O_3_ nanocrystalline	1000	300	12/120	14.29	[39]
Fe-doped SnO_2_ powder	1000	325	-/-	5.88	[40]
5 mol% Pd-SnO_2_	100	260	2.53/9.21	7.78	**This work**
	500		0.83/9.24	18.95	
	1000		0.86/8.63	27.38	
	3000		3.20/6.28	47.58	

*C*, Concentration; OT, Operating temperature; τres/τrec, response/recovery time; β, response.

## Supplementary Materials

The following are available online at http://www.mdpi.com/2079-4991/9/1/13/s1, Figure S1: The N_2_ adsorption-desorption isotherms and the pore size distribution curve (the inset) of (a) pure SnO_2_, (b)–(e) 1, 3, 5 and 7 mol% Pd-functionalized SnO_2_ nanofibers, respectively. Figure S2: XPS spectrums of 5 mol% Pd-functionalized SnO_2_ nanofibers: (a) survey spectrum, (b) Sn 3d spectrum, (c) O 1s spectrum, and (d) Pd 3d spectrum. Figure S3: The reproducibility of 5 mol% Pd-functionalized SnO_2_ nanofibers based sensor on successive exposure to 3000 ppm butane at the optimal operating temperature of 260 °C. Figure S4: The long-term stability of 5 mol% Pd-functionalized SnO_2_ nanofibers based sensor to 3000 ppm butane at 260 °C. Table S1: The information of different content Pd functionalized SnO_2_ samples. Table S2: The concentration ratio of adsorbed oxygen and lattice oxygen.
