# Supplementary material for: Pd-Functionalized SnO2 Nanofibers Prepared by Shaddock Peels as Bio-Templates for High Gas Sensing Performance toward Butane
Source: Nanomaterials (Basel). 2018 Dec 23;9(1):13. doi: 10.3390/nano9010013 (PMC6359564; doi:10.3390/nano9010013)
Supplement: Supplementary file 1 [file nanomaterials-09-00013-s001.pdf]

nanomaterials

MDPI

Supplementary Materials

Pd-Functionalized SnO_2 Nanofibers Prepared by Shaddock Peels as Bio-Templates for High Gas Sensing Performance toward Butane

Rongjun Zhao, Zhezhe Wang, Yue Yang, Xinxin Xing, Tong Zou, Zidong Wang, Ping Hong, Sijia Peng and Yude Wang

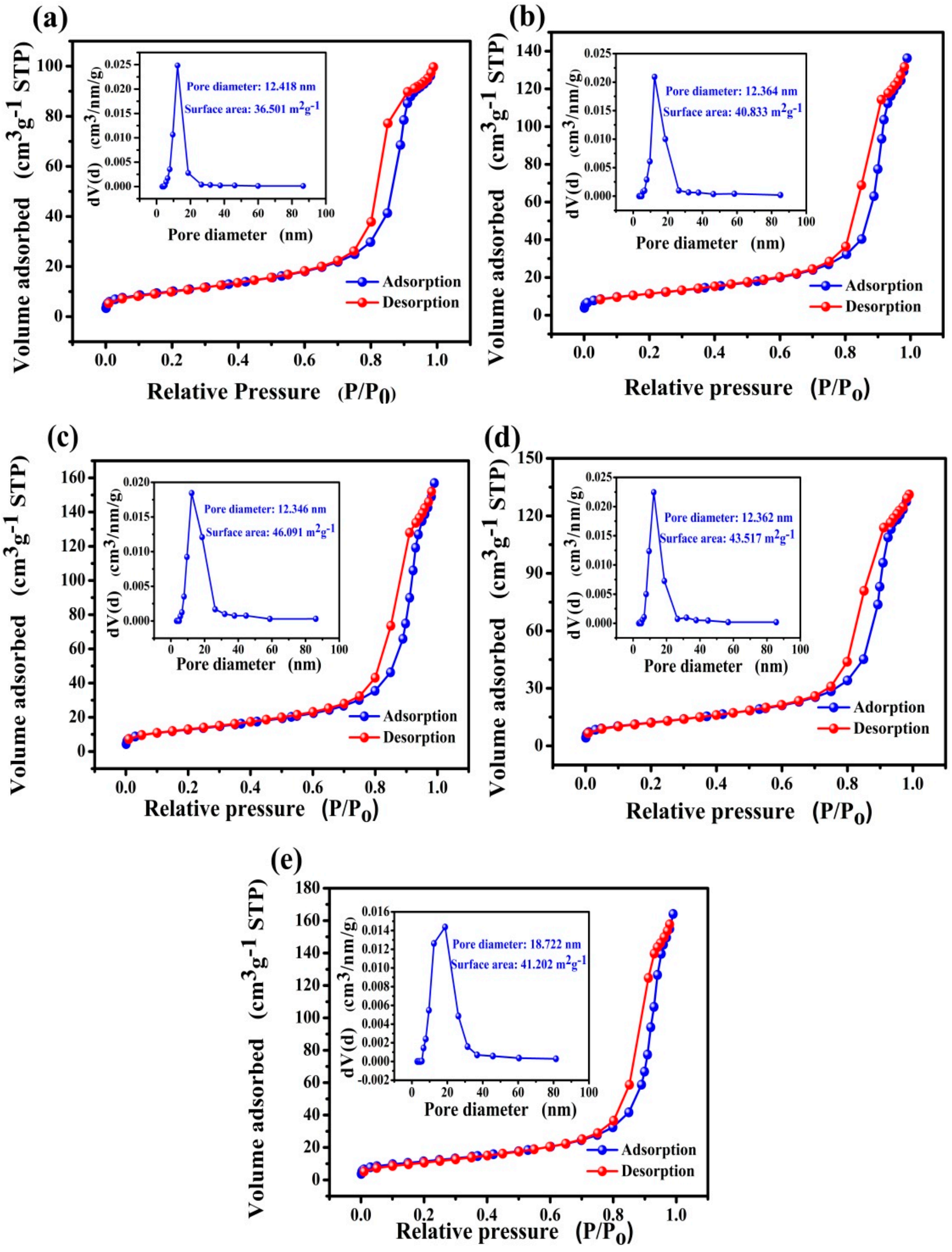

Figure S1. The N_2 adsorption-desorption isotherms and the pore size distribution curve (the inset) of (**a**) pure SnO_2, (**b**)-(**e**) 1, 3, 5 and 7 mol% Pd-functionalized SnO_2 nanofibers, respectively.

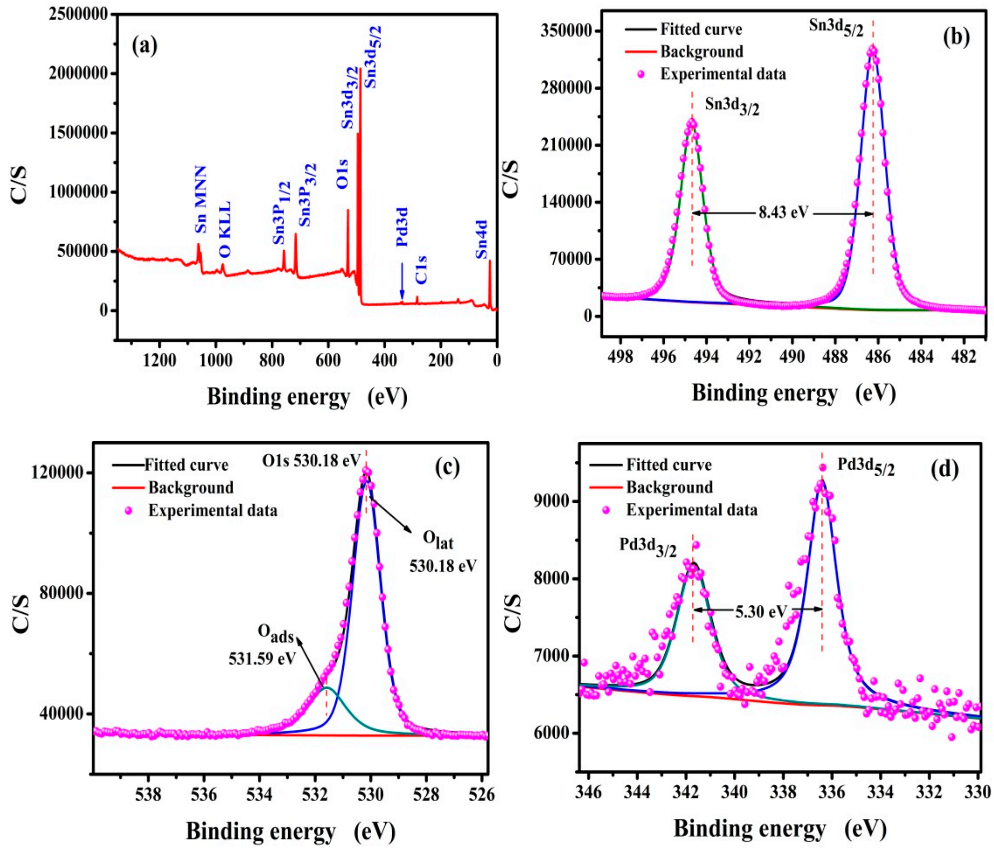

Figure S2. XPS spectrums of 5 mol% Pd-functionalized SnO_2 nanofibers: (**a**) survey spectrum, (**b**) Sn 3d spectrum, (**c**) O 1s spectrum, and (**d**) Pd 3d spectrum.

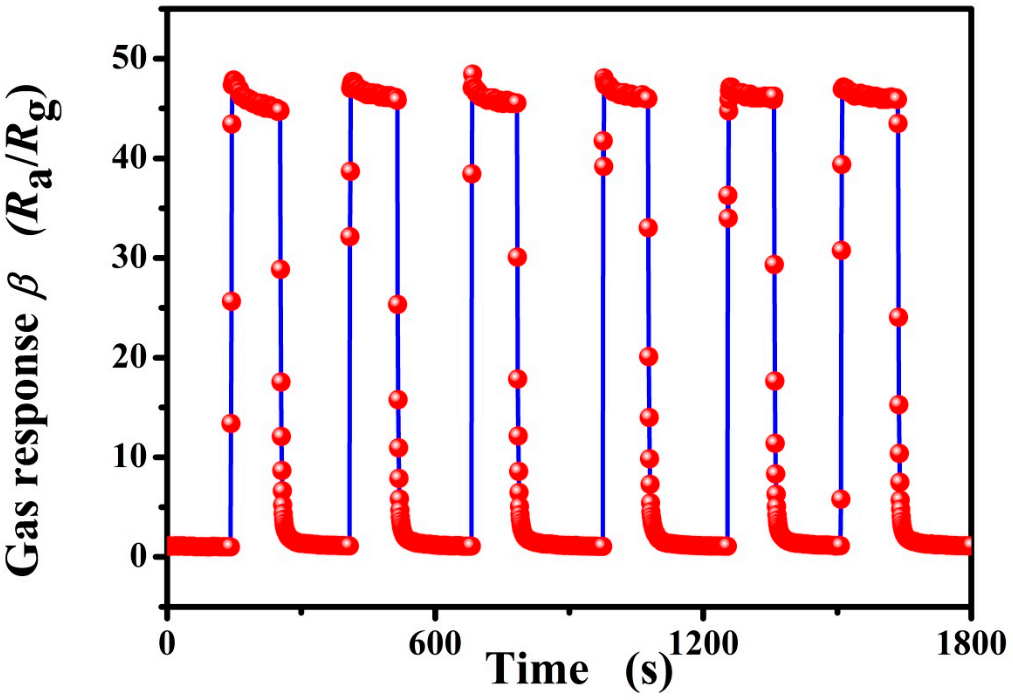

Figure S3. The reproducibility of 5 mol% Pd-functionalized SnO_2 nanofibers based sensor on successive exposure to 3000 ppm butane at the optimal operating temperature of 260 °C.

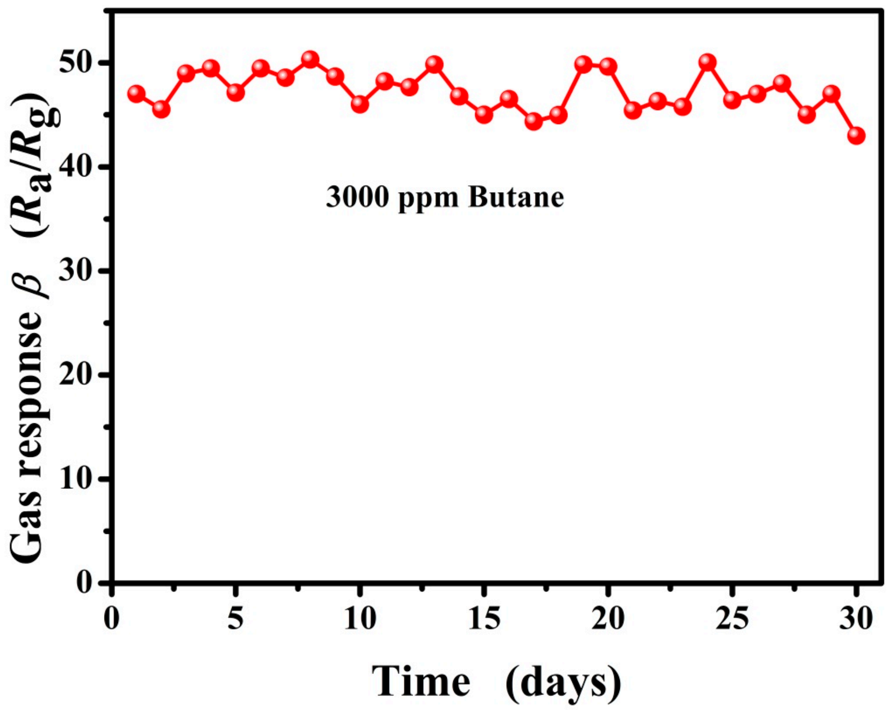

Figure S4. The long-term stability of 5 mol% Pd-functionalized SnO_2 nanofibers based sensor to 3000 ppm butane at 260 ^{o}C.

Table S1. The information of different content Pd functionalized SnO_2 samples.

Sample	SnO_2 crystallite size (Å)	Pd particles size (Å)	Pd content [Pd]/[Sn](mol%)	Specific surface area (m^2/g)
Pristine SnO_2	128	-	-	37
1 mol% Pd-SnO_2	132	154	0.7	41
3 mol% Pd-SnO_2	128	171	2.6	46
5 mol% Pd-SnO_2	132	168	4.5	44
7 mol% Pd-SnO_2	122	159	6.4	41

Table S2. The concentration ratio of adsorbed oxygen and lattice oxygen.

Sample	Adsorbed oxygen (O_{ads})	Lattice oxygen (O_{lat})
Pristine SnO_2	19.4%	80.6%
1 mol% Pd-SnO_2	24.1%	75.9%
3 mol% Pd-SnO_2	30.3%	69.7%
5 mol% Pd-SnO_2	28.9%	71.1%
7 mol% Pd-SnO_2	24.7%	75.3%
